# Insulin-like peptide 5 is released in response to bile acid in the rectum and is associated with diarrhoea severity in patients with bile acid diarrhoea

**DOI:** 10.1136/gutjnl-2025-335393

**Published:** 2025-07-23

**Authors:** Christopher A Bannon, Julian R F Walters, Tongzhi Wu, Richard G Kay, Austin Punnoose, Robin C Spiller, Jonathan Wilson, Petra Verdino, Peter Barker, Keith Burling, Michael Horowitz, Christopher K Rayner, Alexander C Ford, Frank Reimann, Fiona M Gribble

**Affiliations:** 1 Institute of Metabolic Science, Cambridge, UK; 2 Division of Digestive Diseases, Imperial College London, London, UK; 3 Imperial College Healthcare Trust, London, UK; 4 Adelaide Medical School and Centre of Research Excellence in Translating Nutritional Science to Good Health, The University of Adelaide, Adelaide, South Australia, Australia; 5 Nottingham Digestive Diseases Centre, University of Nottingham, Nottingham, UK; 6 NIHR Nottingham Biomedical Research Centre, Nottingham, UK; 7 Eli Lilly and Company, Indianapolis, Indiana, USA; 8 Eli Lilly and Company, Lilly Biotechnology Center, San Diego, California, USA; 9 Leeds Institute of Medical Research at St. James’s, University of Leeds, Leeds, UK; 10 Leeds Gastroenterology Institute, Leeds Teaching Hospitals NHS Trust, Leeds, UK

**Keywords:** DIARRHOEA, GUT HORMONES, IRRITABLE BOWEL SYNDROME, BILE ACID, COLORECTAL MOTILITY

## Abstract

**Background:**

Insulin-like peptide 5 (INSL5) is an enteroendocrine hormone expressed in distal colonic ‘L cells’. Bile acid receptor agonists are known to stimulate INSL5 secretion in primary cell culture, and administration of an INSL5 analogue in animals promotes colonic motility.

**Objective:**

This study used a new immunoassay to measure INSL5 in human blood samples, enabling assessment of whether rectal bile acids stimulate INSL5 release in humans and whether INSL5 levels are altered in patients with chronic diarrhoea.

**Design:**

Serum/plasma samples from previously performed studies were used, including healthy volunteers (n=7) who received a rectal enema of taurocholic acid (TCA); fasting and post prandial samples from healthy volunteers (n=10); patients with bile acid diarrhoea (BAD) (n=19) or irritable bowel syndrome with diarrhoea (IBS-D) (n=8); and patients with IBS-D (n=64) treated with ondansetron or placebo.

**Results:**

Rectal TCA but not a control enema promptly elevated plasma INSL5, with the increase in INSL5 correlating negatively with time to, and positively with desire to, defecate post enema. Healthy volunteers had low INSL5 levels (<100 pg/mL), with no change following a mixed meal. Patients with BAD had elevated INSL5 levels, with average stool consistency being positively correlated with serum INSL5 (p<0.001). In people with IBS-D, INSL5 was elevated (>100 pg/mL) in 42%, and this subgroup showed greater improvements in stool consistency with ondansetron therapy (p<0.05).

**Conclusion:**

The study highlights that rectal bile acids stimulate INSL5 secretion in humans, and that INSL5 levels are associated with a colonic pro-motility response and pathophysiology of chronic diarrhoea.

**WHAT IS ALREADY KNOWN ON THIS TOPIC**

- Insulin-like peptide 5 (INSL5) is a hormone released in the distal colon, from enteroendocrine cells also containing glucagon-like peptide-1 (GLP-1) and peptide YY (PYY).
- Bile acid receptor agonists stimulate co-release of all three hormones in mouse and human primary cultures.
- Stimulation of distal enteroendocrine cells in animal models triggers a defecation response, which is insensitive to inhibition of GLP-1 or PYY receptors, while INSL5 administration promotes colonic bead expulsion in mice.
- Both the defecation response and bead expulsion are attenuated by 5-HT3 (5-hydroxytryptamine type 3) receptor antagonists such as ondansetron.

**WHAT THIS STUDY ADDS**

- This is the first study to explore INSL5 levels in patients with chronic diarrhoea. It highlights that delivery of bile acids to the rectum stimulates INSL5 release and is associated with a defecation response. INSL5 levels are low in healthy volunteers but are raised in patients with bile acid diarrhoea (BAD) and a subset of patients with irritable bowel syndrome with diarrhoea. In patients with BAD, there is a positive correlation between INSL5 levels and stool consistency (measured by Bristol Stool Form Score).

**HOW THIS STUDY MIGHT AFFECT RESEARCH, PRACTICE OR POLICY**

- This study identifies a role for INSL5 in the pathophysiology of chronic diarrhoea and demonstrates that colonic bile acids are one factor that stimulates its release. Further work is needed to identify other local stimulants of INSL5 secretion, to determine the potential utility of measuring INSL5 as an aid to diagnosing conditions involving colonic irritation and whether it is a potential biomarker to predict clinical response to ondansetron in chronic diarrhoea, and to explore the mechanisms underlying links to the pathophysiology of chronic diarrhoea.

## Introduction

 Insulin-like peptide 5 (INSL5) is a relatively recently identified enteroendocrine hormone secreted from distal colonic and rectal enteroendocrine ‘L-cells’ better known for their release of glucagon-like peptide-1 (GLP-1) and peptide YY (PYY).^1^,^3^ Unlike the well-established metabolic roles of GLP-1 and PYY,^1^ the roles of INSL5 in human physiology are poorly characterised, at least in part due to difficulties in measuring circulating INSL5 concentrations.

INSL5 is a member of the relaxin family of peptides.^2^ Its tertiary structure is similar to that of insulin and insulin-like growth factors and it is an agonist of the G protein coupled relaxin/insulin-like family peptide receptor-4 receptor (RXFP4).^2^ Single cell transcriptomic data from human and mouse intestine have highlighted that whereas *INSL5* expression is most enriched in L-cells from the distal colon, *RXFP4* is differentially expressed in colonic 5-hydroxytryptamine (5-HT)-producing enterochromaffin cells.^4 5^ At least in vitro, INSL5 is co-secreted with GLP-1 and PYY in response to a variety of stimuli, including agonists of the G-protein bile acid receptor-1 (GPBAR1) and short chain free fatty acid receptors, as determined using primary epithelial cultures from human or mouse large intestine.^6^

A number of studies in animal models have suggested a role for the Insl5/Rxfp4 axis in regulating colonic motility. Exogenous administration of an INSL5 analogue promoted colonic motility in mice, as measured by the speed of rectal bead expulsion.^7^ Stimulation of *Insl5*-expressing L-cells using a designer receptor exclusively activated by designer drug (DREADD) mouse model induced a defecation response that persisted in the presence of GLP-1 and PYY receptor antagonists.^8^ Antagonism of 5-HT type 3 (5-HT3) receptors, however, blocked both the promotility effects seen in the DREADD mouse model^8^ and the accelerated rectal bead expulsion triggered by exogenous INSL5 analogue.^7^ Whether and how these findings relate to the control of colonic motility in human health and disease is unclear.

Chronic diarrhoea is a common presenting problem in gastroenterology clinics,^9^ with an estimated Western population prevalence of 5%.^10^ Irritable bowel syndrome (IBS) affects up to 5% of the population,^11^ with around one-third of patients with IBS meeting the criteria for IBS with diarrhoea (IBS-D).^12^ Bile acid diarrhoea (BAD) is a common but underdiagnosed condition, with symptoms arising due to excess colonic bile acids.^13^ It is estimated to affect up to one-third of patients with IBS-D, with an estimated population prevalence of 1%.^14^ The SeHCAT test (which assesses whole body retention of ^75^Se-radiolabelled 23-Selena-25-homotaurocholate (tauroselcholate)), is the gold-standard diagnostic test for BAD in the UK.^13^ Other diagnostic tests for BAD include measurement of faecal bile acids^15^ and serum concentrations of the bile acid precursor 7α-OH-4-cholesten-3-one (C4) as a marker of the rate of hepatic bile acid production.^16^ Secondary BAD results from decreased intestinal reabsorption of bile acids, which in healthy individuals occurs in the distal ileum through the activity of apical sodium coupled bile acid transporters but is disrupted following ileal resection or in other gastrointestinal disorders affecting the ileum, including Crohn’s disease or pelvic radiotherapy.^13^ Primary BAD is idiopathic and associated with overproduction of bile acids.^13 17 18^

Patients with BAD and IBS-D report symptoms including urgency, increased stool frequency and faecal incontinence.^12 13^ Treatment regimens differ, with IBS-D pharmacotherapy aimed at reducing bowel frequency using agents such as loperamide^19^ or the 5-HT3 receptor inhibitor ondansetron.^20^ Principal treatment for BAD is the use of bile acid sequestrants,^21^ but recent evidence also shows symptom improvement with the GLP-1 receptor agonist liraglutide.^22^

Although circulating INSL5 concentrations have been measured previously in a small number of clinical studies, outcomes were inconclusive. While these studies reported a decrease in plasma INSL5 following weight loss post bariatric surgery^23^ and elevated serum levels in patients with polycystic ovarian syndrome,^24^ the reported INSL5 concentrations were several orders of magnitude higher than typical levels of the co-secreted hormones GLP-1 and PYY, raising questions about assay reliability. No studies to date have assessed INSL5 levels in patients with gastrointestinal disease, despite its association with promoting colonic motility in animal studies.

Based on the known stimulation of INSL5 release in vitro by GPBAR1 agonists, we investigated circulating INSL5 levels in healthy human volunteers given rectal bile acid enemas, alongside INSL5 levels in patients with BAD, and patients with IBS-D from previously performed studies.

## Methods

### INSL5 measurements by immunoassay

A new INSL5 sandwich immunoassay based on two anti-INSL5 antibodies was developed, which had a sensitivity limit of~50 pg/mL. To validate the assay, we confirmed that the capture antibody immunoprecipitated exogenous INSL5 spiked into human plasma, which was detectable by liquid chromatography-tandem mass spectrometry (LC-MS/MS) (online supplemental figure 1).

### Samples from previously performed studies

INSL5 was measured via immunoassay in samples from previously performed studies, including:

1. Stored plasma samples from seven healthy volunteers who received a blinded rectal perfusion of a 20 mL enema containing either 1500 mg or 3500 mg taurocholic acid (TCA) or vehicle (placebo) after an overnight fast in a double-blind, randomised, crossover fashion.^25^
2. Stored plasma samples from 10 healthy volunteers with no prior history of gastrointestinal or endocrine illness; fasting and up to 2 hours after a liquid mixed meal,^26^ 8 of whom had bowel habit diary data.
3. Stored serum samples from patients with primary BAD (n=9), secondary BAD (n=10) and IBS-D (n=8); fasting and at time points 1 and 3 hours after a solid mixed meal. Patients withheld bile acid sequestrants for 14 days prior to the visit, and the samples are from the first visit of the study before administration of obeticholic acid.^27^
4. Stored serum samples from patients with IBS-D recruited into the TRITON study. 65 samples were available, of which one was excluded due to suspected assay interference/analytical error. 62 participants had available bowel habit diary data at baseline before receiving placebo or ondansetron,^20^ and 50 had bowel habit data at baseline and for the final week of placebo or ondansetron treatment.

Bowel habit diaries were available from the previous studies for 7–14 days before blood samples were taken, from which metrics were extracted of average stool consistency as measured by Bristol Stool Form Score (BSFS). Samples from Wu et *al* had been previously assayed for total GLP-1 and PYY,^25^ alongside a recorded time to defecate post enema and measures of desire to defecate at each time point, taken from a 10 cm Visual Analogue Scale.

From the study of Walters et *al*,^27^ all patients with IBS-D had SeHCAT test results of>15% retention, indicating absence of BAD. Participants from this study had been allowed to take loperamide, with documented as required doses included in the bowel habit diary to allow generation of a calculated diarrhoea index score.^27^ Previous biochemistry measurements on the samples included C4 via LC-MS/MS.^27^

From the TRITON study,^20^ patients aged ≥18 years were required to meet Rome IV criteria for IBS-D and to have had other likely causes of diarrhoea excluded, including microscopic colitis, BAD, coeliac disease or lactose intolerance, with full eligibility described previously.^20^ Patients in the TRITON study had BAD excluded by SeHCAT retention>10% or serum C4, other than four patients at one site who had a 1 week therapeutic trial of a bile acid sequestrant without symptom improvement.^20 28^ In the 53 participants who provided a stool sample in the TRITON study, faecal bile acid levels were normal and did not meet criteria for BAD.^20^ Previously measured faecal bile acid metabolites as mg/g dry stool were available for 44 participants at baseline.

Summaries of previous study demographics are given in table 1.

**Table 1:** Summary of demographics of samples included from previously performed studies

Diagnosis	Study reference	N	Gender	Age	BMI
Healthy volunteer	25	7	7 M	40.0 (14.1)	27.0 (1.8)
Healthy volunteer	26	10	7 M, 3 F	38.6 (11.1)	24.5 (2.7)
Primary BAD	27	9	3 M, 6 F	46.1 (16.4)	26.8 (5.8)
Secondary BAD	27	10	3 M, 7 F	48.2 (15.9)	24.5 (2.9)
IBS-D—Walters *et al*	27	8	5 M, 3 F	40.0 (12.9)	26.1 (5.8)
IBS-D—TRITON study	20	64	26 M, 38 F	43.6 (15.9)	Not recorded

BAD, bile acid diarrhoea; BMI, body mass index; F, female; IBS-D, irritable bowel syndrome with diarrhoea; M, male; TRITON, abbreviated name of previously performed clinical trial.

### Patient and public involvement

As the study featured samples from previously performed studies, patients and the public were not involved in the design and plans for this research.

### Ethics

This study involves research on previously collected samples, with ethical approval obtained prior to each previous study.

The protocol for collecting samples from healthy volunteer samples^26^ was reviewed by East of Scotland Research Ethics Service (22/ES/0021) sponsored by Cambridge University Hospitals NHS Foundation Trust and University of Cambridge. The protocol for collecting samples from patients with chronic diarrhoea^27^ was reviewed by National Research Ethics Service Committee London Brent (12/LO/0123) and was an investigator-initiated trial, known as OBADIAH1, sponsored by Imperial College London and Imperial College Healthcare NHS Trust and registered with EudraCT (2011-003777-28) and ClinicalTrials.gov (NCT01585025). The protocol for the TRITON Study^20^ was reviewed by Yorkshire and The Humber - Leeds West Research Ethics Committee (17/YH/0262) and was registered with EudraCT (2017-000533-31) and registered with an International Standard Randomised Controlled Trial Number (ISRCTN17508514), and was sponsored by Nottingham University Hospitals NHS trust. The protocol for collecting samples from volunteers receiving rectal enema^25^ was reviewed by the Human Research Ethics Committee of the Royal Adelaide Hospital, South Australia.

### INSL5 measurements by LC-MS/MS

LC-MS/MS quantification of INSL5 in human serum used a modified method described previously for analysis of INSL5 in tissues.^29^ In brief, serum samples were precipitated using acetonitrile, and reduction and alkylation were performed to break disulphide bonds before LC-MS/MS analysis, with spiked-in bovine insulin used as an internal standard to compensate for procedural losses. A calibration line of human INSL5 (Phoenix Pharmaceuticals, Burlingame, USA) was prepared from 100 to 2000 pg/mL and extracted alongside samples. The lower limit of detection of the reduced and alkylated INSL5 B-chain was determined as 100 pg/mL.

### Additional measurements on samples via immunoassay

Serum samples from Walters et *al*^27^ were assayed for PYY, GLP-1 and INSL5 (table 2). Plasma samples from Foreman et *al*^26^ were analysed for fasting serum PYY and INSL5. A subset of available TRITON serum samples^20^ was analysed for PYY (n=51) and INSL5 (n=64).

**Table 2:** Summary of immunoassays performed on previous study samples

Analyte	Assay	Product code	Supplier
INSL5	In-house immunoassay	N/A	Antibodies from Eli Lilly
GLP-1	Total GLP-1 V.2	K150JVC-4	Mesoscale Discovery
PYY	U-plex Total PYY	K1516BK	Mesoscale Discovery

GLP-1, glucagon-like peptide-1; INSL5, insulin-like peptide 5; PYY, peptide YY.

### Statistical tests and data visualisation

Statistical analysis and data visualisation were performed in Visual Studio Code using python V.3.13.0 using the packages pandas, numpy, statsmodels, seaborn, matlabplotlib, scipy and scikit-learn. Significance levels were set at 5% (p<0.05). Data are shown as mean and SD, or median and IQR if normality could not be assumed (via Shapiro-Wilk test).

For time series samples from meal or enema studies, fasting or pre-enema data are shown, alongside post-meal and post-enema values as mean and SE. Swarm plots with individual data points are used to present individual INSL5 levels between groups, with dotted lines marking the immunoassay sensitivity cut-off of 50 pg/mL.

For the enema study, where the same participants received different doses at each visit, a one-way repeated measures analysis of variance (ANOVA) model was used to compare the incremental area under the curve (iAUC) of INSL5 in response to different doses of TCA, with iAUC calculated using the trapezoid rule.

To compare INSL5 levels between different patient groups, an analysis of covariance (ANCOVA) model was used with age, body mass index and gender as covariates. Tukey’s post hoc testing for multiple comparisons was performed if the model was significant (p<0.05).

For assessing the treatment effect of ondansetron on BSFS at week 12 in the IBS-D TRITON study, an ANCOVA model was used with BSFS at week 12 as the dependent variable, treatment allocation (active vs placebo) and INSL5 group (above or below threshold) as categorical variables and baseline BSFS at enrolment and interaction between treatment allocation and INSL5 group as covariates. Different thresholds of INSL5 from 50 to 350 pg/mL were used in a sensitivity analysis, assessing for a cut-off with an interaction between INSL5 group and treatment allocation with the lowest p value. The same analysis was performed for PYY using steps of 10 pg/mL.

Correlations are reported as p value and r^2^ alongside scatterplots. For modelling purposes, INSL5 concentrations below the limit of detection (50 pg/mL for immunoassay and 100 pg/mL for LC-MS/MS method) were assigned a value of 25 pg/mL. For all statistical tests, normality was assessed by Shapiro-Wilk test (p>0.05), alongside inspection of Q-Q plots and histograms of data points and residuals. If normality could not be assumed, natural logarithm transformation was performed.

## Results

### Rectal delivery of taurocholic acid leads to prompt elevation in INSL5 and stool urgency

**Figure 1:**
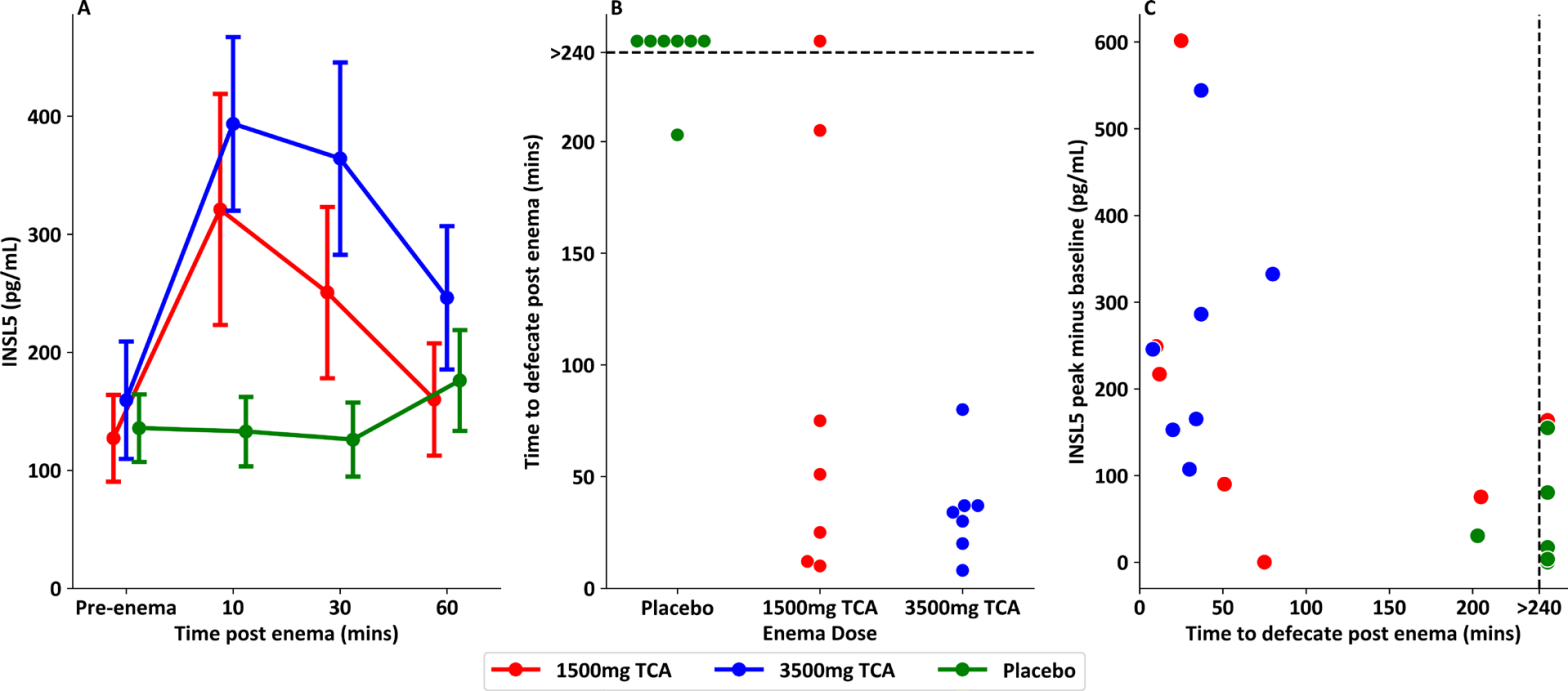
INSL5 levels after blinded placebo or taurocholic acid (TCA) containing enema. (**A**) INSL5 responses in seven healthy volunteers receiving blinded enema on separate visits of placebo, 1500 mg TCA or 3500 mg TCA. One-way repeated measures ANOVA showed a significant effect of dose of TCA on INSL5 iAUC (*F*(2,12)=8.50, p=0.005). (**B**) Swarm plot showing different time to defecate following enema, recorded until 4 hours after enema. Dotted line shows cut-off, as defecation times were collected until 4 hours post enema. (**C**) Scatterplot showing the delta between peak INSL5 level post enema and pre-enema INSL5 level versus the time to defecate post enema. Linear regression performed to compare INSL5 delta versus time to defecate, showing evidence of a negative correlation (p=0.005, r^2^=0.34). Samples from Wu *et al.*^25^ ANOVA, analysis of variance; iAUC, incremental area under the curve; INSL5, insulin-like peptide 5.

Volunteers receiving 1500 mg or 3500 mg TCA by rectal enema, but not placebo enema, showed a twofold to threefold elevation of INSL5 after 10 min, with concentrations declining over the following hour (figure 1A). One way repeated measure ANOVA analysis of INSL5 iAUC showed a significant effect of enema dose (*F*(2,12)=8.50, p=0.005). Pre-enema and vehicle control INSL5 concentrations were towards the lower level of detection of the immunoassay. A subset of samples from the 3500 mg TCA enema was additionally analysed using an LC-MS/MS method for INSL5; post-enema concentrations measured by LC-MS/MS were similar to those measured by immunoassay, providing additional validation of the immunoassay method (online supplemental figure 2).

All volunteers defecated within 100 min of the enema containing 3500 mg TCA, compared with 5/7 who received 1500 mg TCA and none who received placebo (figure 1B). The increment between pre-enema INSL5 and peak post-enema INSL5 showed a negative linear correlation with time to defecate post-enema (figure 1C, p=0.005, r^2^=0.34), with significant evidence also of negative correlations between absolute peak INSL5 levels or INSL5 iAUC and time to defecate (online supplemental figure 3). INSL5 concentrations at each time point were positively correlated with corresponding desire to defecate (online supplemental figure 4, p<0.001, r^2^=0.16), consistent with the concept that INSL5 promotes faecal expulsion.

**Figure 2:**
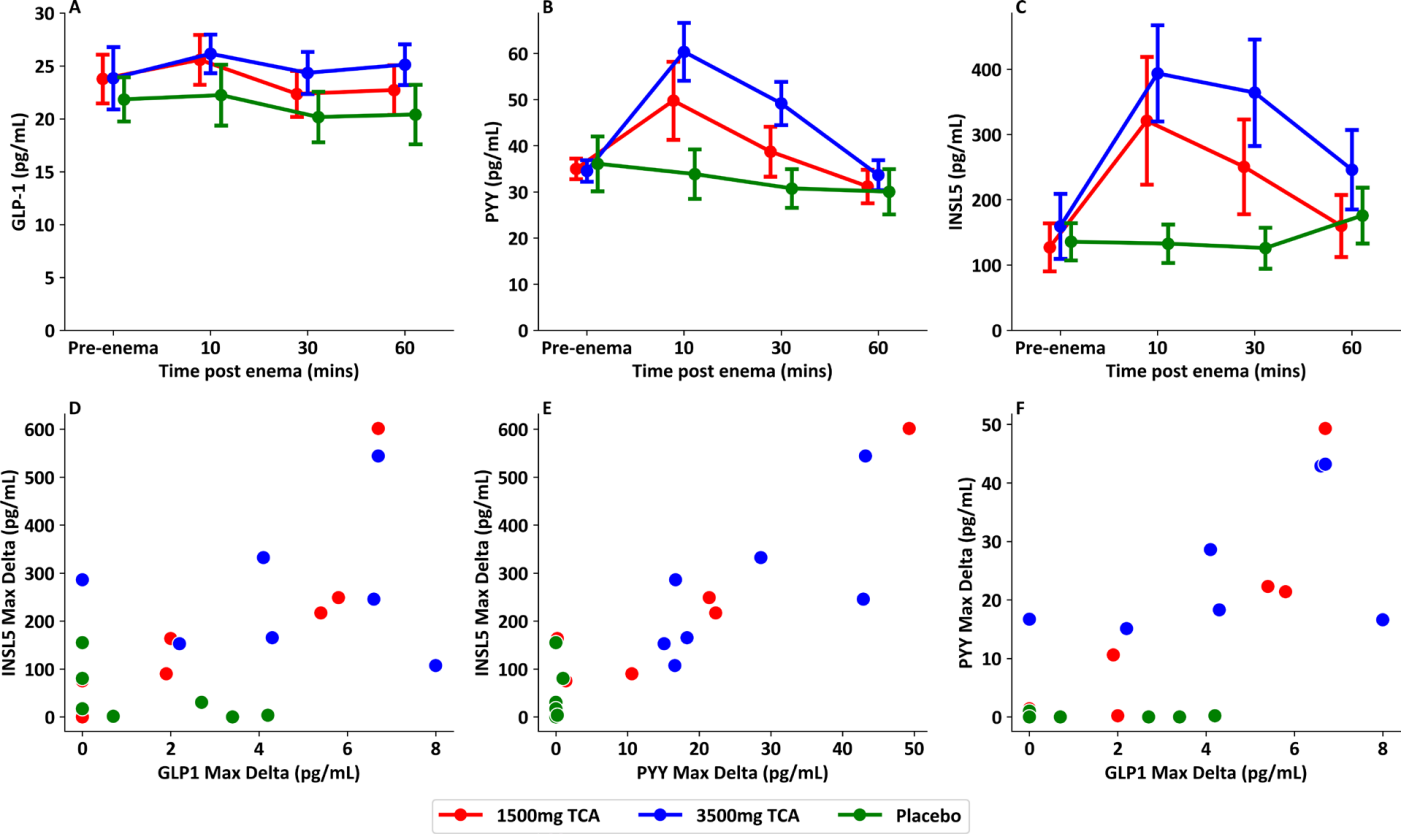
Comparison in L cell hormone levels following taurocholic acid (TCA) containing enema. (**A**) Total GLP-1 responses measured previously in seven healthy volunteers receiving blinded enema on separate visits of placebo, 1500 mg TCA or 3500 mg TCA (data from Wu *et al*^25^). (**B**) PYY responses measured previously in seven healthy volunteers receiving blinded enema on separate visits of placebo, 1500 mg TCA or 3500 mg TCA. (**C**) INSL5 responses in seven healthy volunteers receiving blinded enema on separate visits of placebo, 1500 mg TCA or 3500 mg TCA. (**D**) Scatterplot showing difference between baseline and peak INSL5 versus difference between baseline and peak total GLP-1 responses, (p=0.011, r^2^=0.30). (**E**) Scatterplot showing difference between baseline and peak INSL5 versus difference between baseline and peak PYY responses, (p<0.001, r^2^=0.79). (**F**) Scatterplot showing difference between baseline and peak PYY versus difference between baseline and peak total GLP-1 responses, (p<0.001, r^2^=0.55). Samples from Wu *et al.*^25^ GLP-1, glucagon-like peptide-1; INSL5, insulin-like peptide 5; PYY, peptide YY.

Peak INSL5 increments in response to the enema correlated with peak PYY increments across all TCA doses (figure 2, p<0.001, r^2^=0.79), and weakly correlated with GLP-1 increments (figure 2, p=0.011, r^2^=0.30).

### INSL5 levels are raised in patients with BAD and correlate with Bristol Stool Form Scores and serum PYY

**Figure 3:**
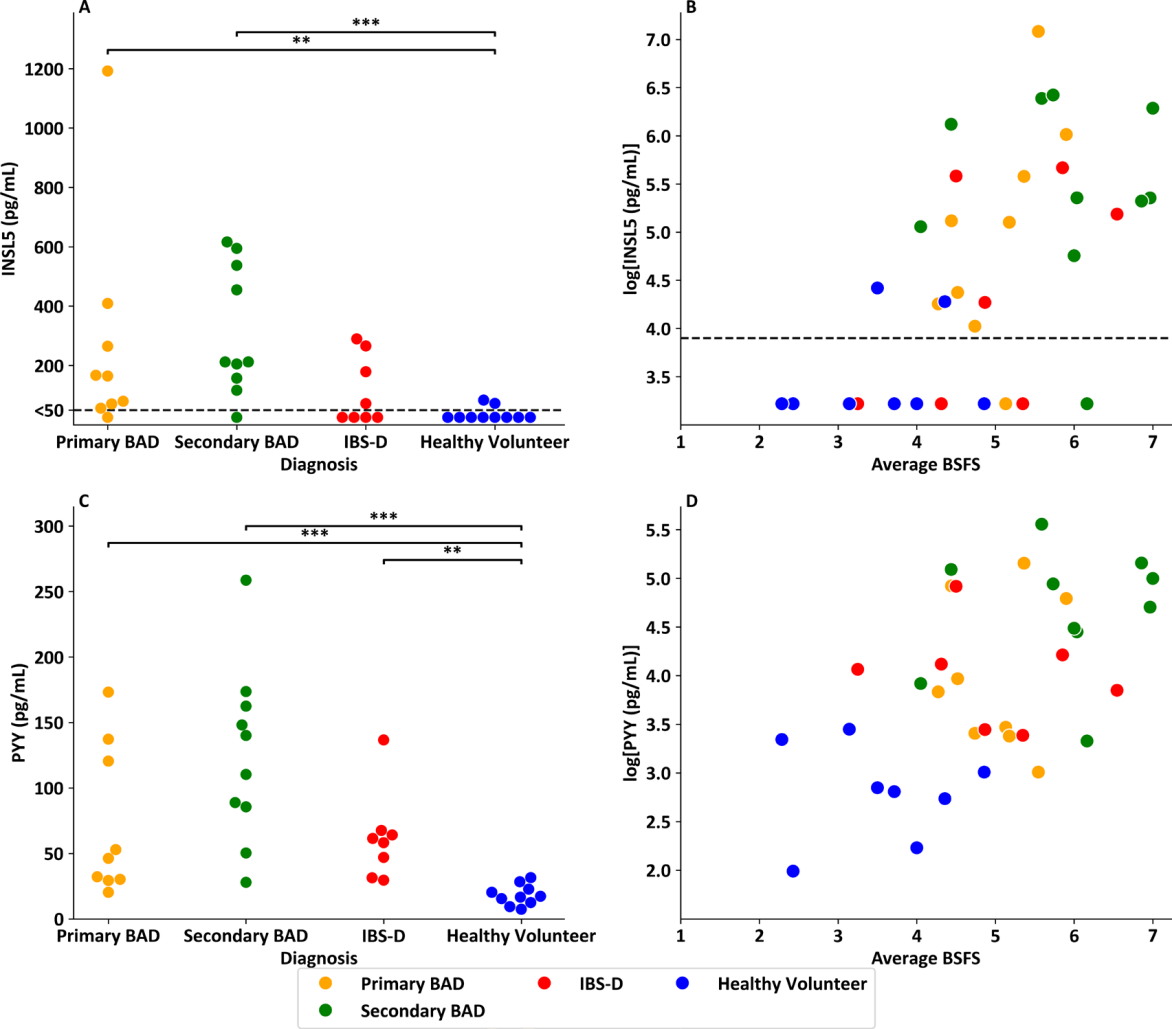
INSL5 and total PYY levels in patients with BAD. (**A**) Swarm plot showing fasting INSL5 levels in healthy volunteers (n=10), patients with primary BAD (n=9), secondary BAD (n=10) and SeHCAT negative IBS-D (n=8). Dotted line denotes immunoassay sensitivity limit of 50 pg/mL. An ANCOVA model performed on natural logarithm transformed data with age, BMI and gender as covariates showed a significant main effect of diagnosis (*F*(3,30)=7.01, p=0.001). Tukey HSD test for multiple comparisons on natural logarithm transformed data showed statistically significant differences between primary BAD and healthy volunteers (p=0.008) and secondary BAD and healthy volunteers (p<0.001). (**B**) Scatterplot showing natural logarithm transformed INSL5 levels versus average BSFS from bowel habit chart (p<0.001, r^2^=0.31)—(note healthy volunteers n=8 as two did not return bowel habit diaries). Healthy volunteer samples (n=10) from Foreman *et al*,^26^ and IBS-D (n=8) and BAD (n=18) samples from Walters *et al*.^27^ (**C**) Swarm plot showing fasting PYY levels in healthy volunteers (n=10), patients with primary BAD (n=9), secondary BAD (n=10) and SeHCAT negative IBS-D (n=8). Dotted line denotes immunoassay sensitivity limit of 50 pg/mL. An ANCOVA model performed on natural logarithm transformed data with age, BMI and gender as covariates showed a significant main effect of diagnosis (*F*(3,30) = 18.7, p<0.001). Tukey HSD test for multiple comparisons on natural logarithm transformed data showed statistically significant differences between primary BAD and healthy volunteers (p<0.001); secondary BAD and healthy volunteers (p<0.001) and IBS-D and healthy volunteers (p=0.001). (**D**) Scatterplot showing natural logarithm transformed PYY levels versus average BSFS from bowel habit chart (p=0.001, r^2^=0.29)—(note, healthy volunteers n=8 as two did not return bowel habit diaries). Healthy volunteer samples (n=10) from Foreman *et al*,^26^ and IBS-D (n=8) and BAD (n=19) samples from Walters *et al*.^27^ ANCOVA, analysis of covariance; BAD, bile acid diarrhoea; BMI, body mass index; BSFS, Bristol Stool Form Scale; IBS-D, irritable bowel syndrome with diarrhoea; INSL5, insulin-like peptide 5; PYY, peptide YY; Tukey HSD, Tukey Honestly Significant DIfference Test; SeHCAT test, assesses whole body retention of ^75^Se-radiolabelled 23-Selena-25-homotaurocholate (tauroselcholate) to diagnose bile acid diarrhoea.

In healthy volunteers with no history of endocrine or gastrointestinal disease (n=10 from Foreman *et al*^26^), fasting serum INSL5 levels were low (<100 pg/mL), in many cases below the 50 pg/mL limit of the immunoassay (figure 3A). In samples from Walters et *al*,^27^ fasting INSL5 concentrations>100 pg/mL were observed in~90% of patients with BAD (8/9 with primary BAD and 9/10 with secondary BAD) and 50% (4/8) of patients with IBS-D with confirmed negative SeHCAT.^27^ INSL5 levels were significantly increased in patients with primary BAD (adjusted p value (p_adj_)=0.008) and secondary BAD (p_adj_<0.001) compared with healthy volunteers. Fasting INSL5 levels had a linear relationship with average BSFS across this sample set including healthy volunteers (n=8), patients with BAD (n=18) and patients with SeHCAT negative IBS-D (n=8) (figure 3B, p<0.001, r^2^=0.33), with participants with higher INSL5 levels more likely to have a looser, more watery stool type.

**Figure 4:**
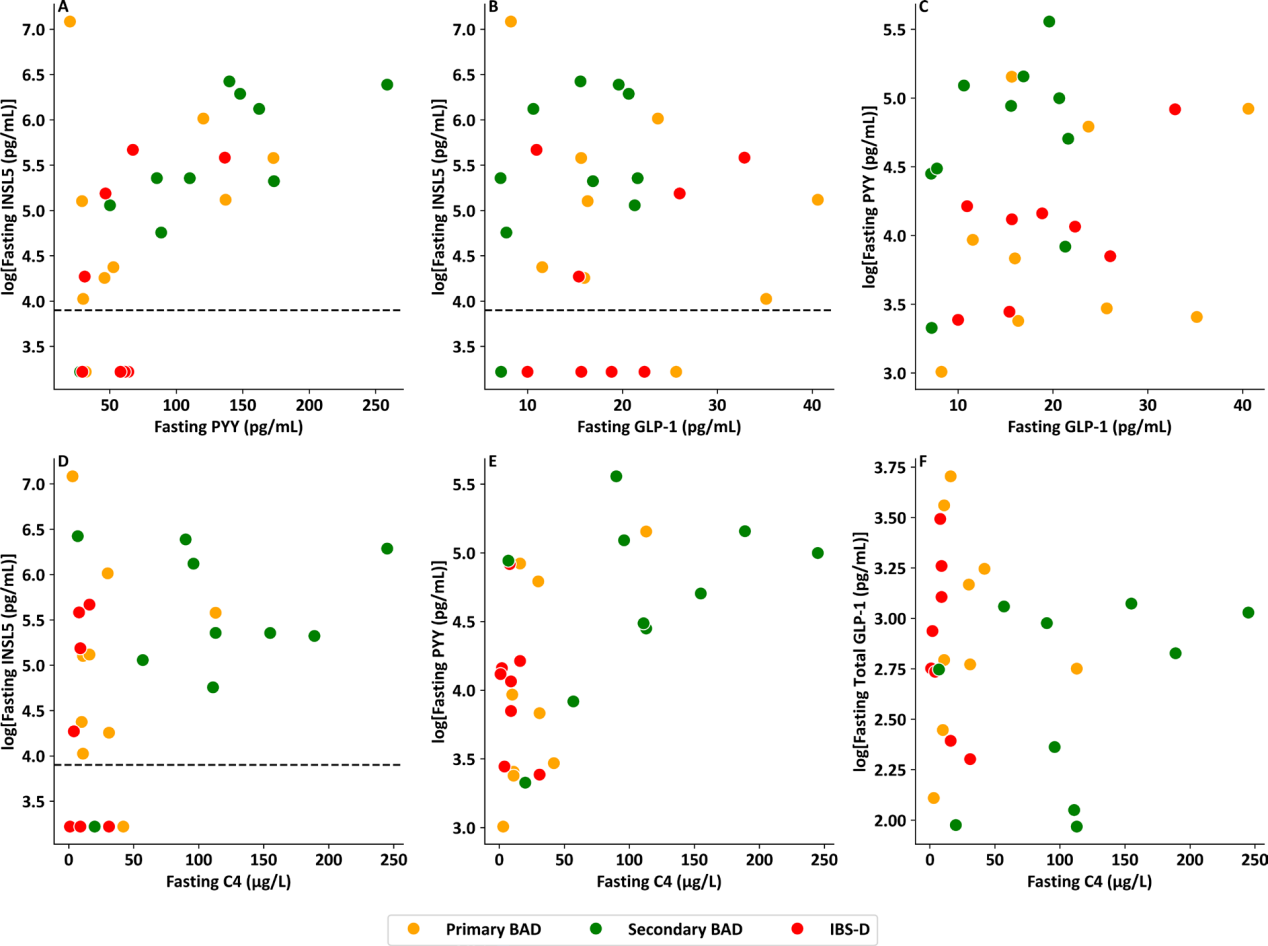
Scatterplots comparing fasting L cell hormones and C4. (**A**) Scatterplot of log transformed fasting serum INSL5 level versus fasting serum PYY in patients with chronic diarrhoea (p=0.002, r^2^=0.33). (**B**) Scatterplot of log transformed fasting serum INSL5 level versus fasting serum total GLP-1 in patients with chronic diarrhoea (p=0.876, r^2^<0.01). (**C**) Scatterplot of log transformed fasting serum PYY level versus fasting serum total GLP-1 in patients with chronic diarrhoea (p=0.368, r^2^=0.03). (**D**) Scatterplot of log transformed fasting serum INSL5 level versus fasting serum C4 in patients with chronic diarrhoea (p=0.063, r^2^=0.13). (**E**) Scatterplot of log transformed fasting serum PYY level versus fasting serum C4 in patients with chronic diarrhoea (p=0.002, r^2^=0.32). (**F**) Scatterplot of log transformed fasting serum total GLP-1 level versus fasting serum C4 in patients with chronic diarrhoea (p=0.661, r^2^=0.01). Note: all samples from Walters *et al*.^27^ BAD, bile acid diarrhoea; GLP-1, glucagon-like peptide-1; IBS-D, irritable bowel syndrome with diarrhoea; INSL5, insulin-like peptide 5; PYY, peptide YY.

PYY was similarly significantly elevated in patients with primary BAD (p_adj_<0.001), secondary BAD (p_adj_<0.001) and IBS-D (p_adj_=0.001) compared with healthy volunteers (figure 3C). Correlation analysis also revealed a significant association between fasting PYY and BSFS (figure 3D, p=0.001, r^2^=0.29). INSL5 correlated significantly with PYY (p=0.002, r^2^=0.33) but not GLP-1 (p=0.88) (figure 4A–C). There was a significant relationship between C4 and PYY (p=0.002, r^2^=0.32), a trend for a significant relationship between C4 and INSL5 (p=0.063, r^2^=0.13) and no relationship between C4 and GLP-1 (p=0.661) (figure 4D–F).

### INSL5 levels do not change in response to nutrient intake

**Figure 5:**
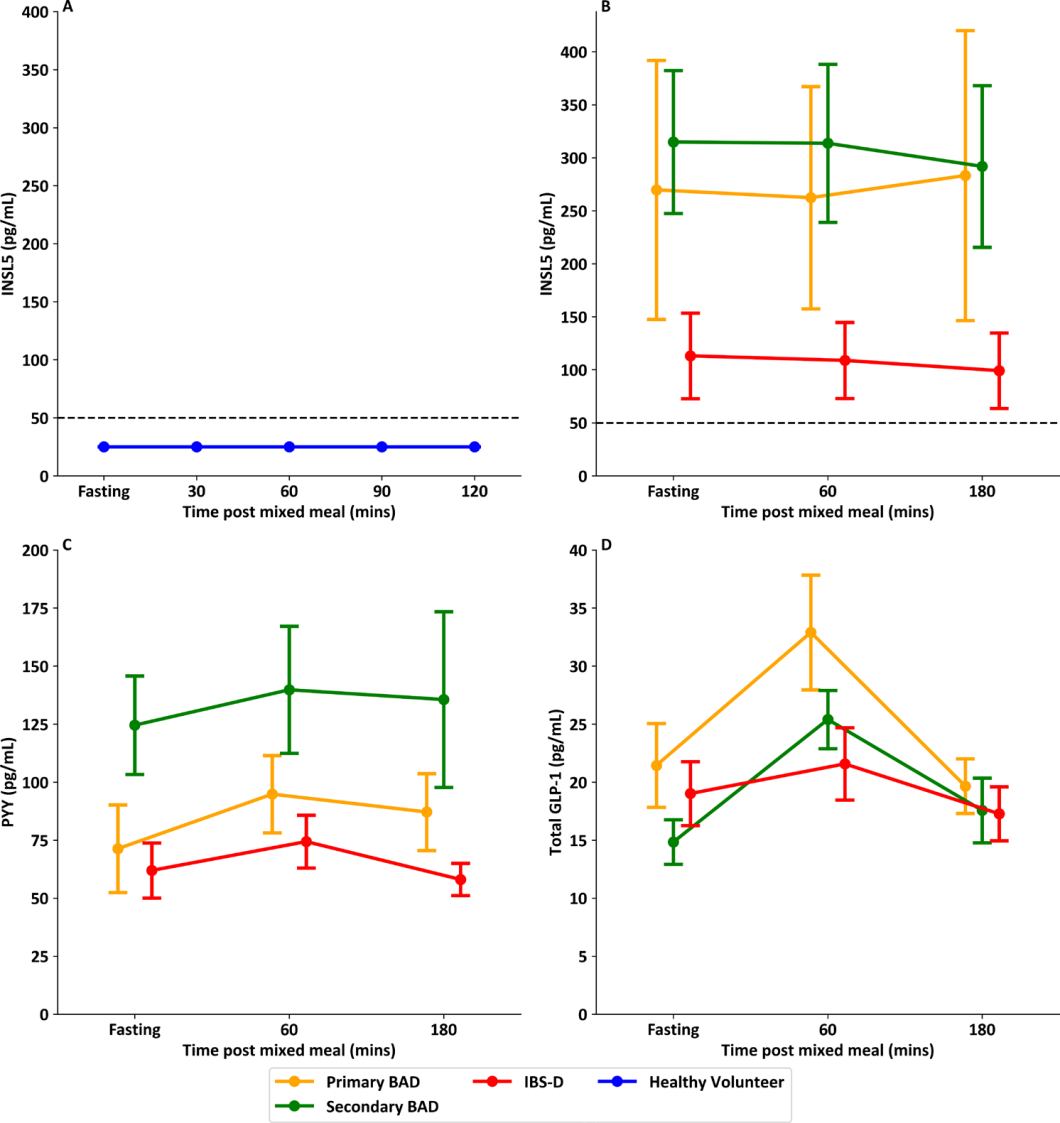
Post-prandial INSL5 levels compared with post-prandial PYY and total GLP-1 levels. (**A**) Point plot showing levels of INSL5 before and after a liquid mixed meal in healthy volunteers (mean and SEM). (**B**) Point plot showing levels of INSL5 before and after a solid mixed meal in patients with chronic diarrhoea (mean and SEM). (**C**) Point plot showing levels of PYY before and after a meal in patients with chronic diarrhoea (mean and SEM). (**D**) Point plot showing levels of total GLP-1 before and after a meal in patients with chronic diarrhoea (mean and SEM). Healthy volunteer samples (n=8) from Foreman *et al*,^26^ and IBS-D (n=8) and BAD (n=18) samples from Walters *et al*.^27^ BAD, bile acid diarrhoea; GLP-1, glucagon-like peptide-1; IBS-D, irritable bowel syndrome with diarrhoea; INSL5, insulin-like peptide 5; PYY, peptide YY.

There was no measurable change in INSL5 in healthy volunteers in response to a liquid mixed meal, with all levels remaining below the immunoassay detection limit (figure 5A). There was also no evidence of a change in INSL5 in patients with chronic diarrhoea who ate a solid mixed meal, including in patients with higher INSL5 levels in the fasting state (figure 5B). Patients with chronic diarrhoea did, however, show expected postprandial increases in PYY and GLP-1 1 hour post solid mixed meal (figure 5C,D).

### INSL5 levels are raised in a proportion of patients with IBS-D, identifying a patient subset showing improved ondansetron responses

**Figure 6:**
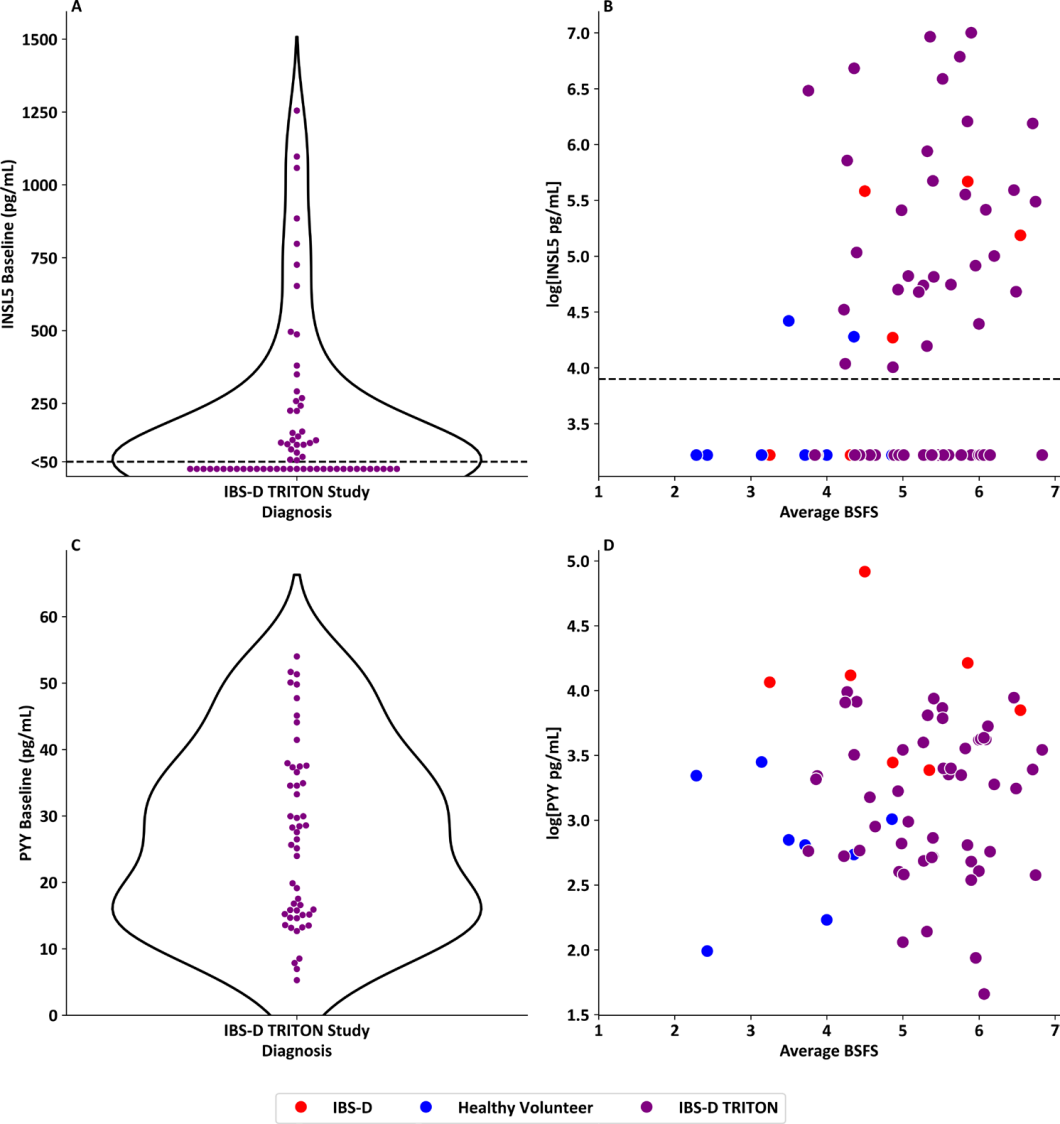
INSL5 and PYY levels in patients with IBS-D from the TRITON study. (**A**) Violin plot of baseline INSL5 levels in patients with IBS-D recruited into the TRITON study, highlighting a distribution where 50% of samples have a detectable INSL5>50 pg/mL and 42% of patients with IBS-D had an INSL5 level>100 pg/mL (n=64). (**B**) Scatterplot of natural log transformed serum INSL5 level versus average BSFS in healthy volunteers (n=8) (from Foreman *et al*^26^ and patients with IBS-D (n=66) (from both TRITON study (n=62, purple dots)^20^ and Walters *et al*^27^ (n=8, red dots)), (p=0.033, r^2^=0.06). (**C**) Violin plot of baseline PYY levels in patients with IBS-D recruited into the TRITON study, (n=51). (**D**) Scatterplot of natural log transformed serum PYY level versus average BSFS in healthy volunteers (n=8) (from Foreman *et al*^26^ and patients with IBS-D (n=66) (from both TRITON study (n=51, purple dots)^20^ and Walters *et al*^27^ (n=8, red dots)), (p=0.716). BSFS, Bristol Stool Form Scale; IBS-D, irritable bowel syndrome with diarrhoea; INSL5, insulin-like peptide 5; PYY, peptide YY; TRITON study, abbreviation for previously performed clinical trial.

In baseline IBS-D samples from the TRITON study (n=64),^20^ there was a wide range in INSL5 levels (median<50 pg/mL, (IQR:<50–224.1 pg/mL)), with 50% of samples having values above the detection limit 50 pg/mL and 42% being>100 pg/mL (figure 6A). For patients with IBS-D from both the TRITON study (n=62) and Walters et *al* (n=8) study, and healthy volunteers^26^ (n=8) with bowel habit data, there was some evidence of correlation between INSL5 and average stool consistency from BSFS (p=0.033, r^2^=0.06) (figure 6B). By contrast, there was no evidence of correlation between BSFS and PYY levels in patients with IBS-D, combining data from the TRITON study (figure 6C), Walters *et al* and healthy volunteers (p=0.716) (figure 6D).

In the TRITON group, baseline faecal bile acids were available for 44 participants. Serum INSL5 was significantly correlated with the dry mass of lithocholic acid (p=0.029, r^2^=0.11, n=89), but not with other bile acids or total stool bile acids (online supplemental figure 5).

**Figure 7:**
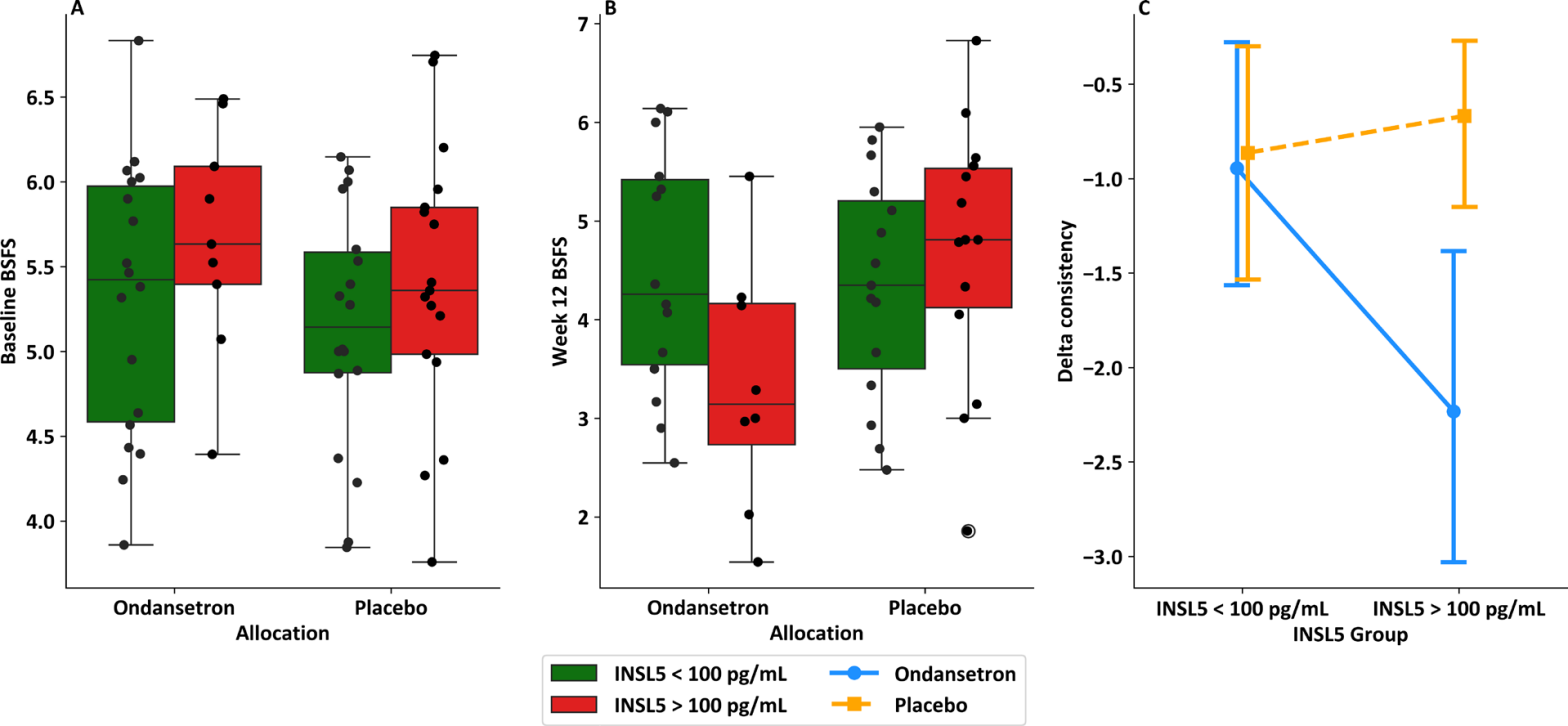
Patients with IBS-D with a raised INSL5 level from the TRITON study showed a greater improvement in stool consistency in response to ondansetron. (**A**) Box plot with individual data points showing BSFS at baseline (n=62) in each treatment group (placebo n=35 and ondansetron n=27), and highlighting participants in each group per INSL5 group (placebo: INSL5>100 pg/mL n=17, INSL5<100 pg/mL n=18; ondansetron: INSL5>100 pg/mL n=9, INSL5<100 pg/mL n=18); (**B**) Box plot with individual data points showing BSFS after 12 weeks of treatment (n=51) in each treatment group (placebo n=29 and ondansetron n=22), and highlighting participants in each group per INSL5 group (placebo, INSL5>100 pg/mL n=14, INSL5<100 pg/mL n=15; ondansetron, INSL5>100 pg/mL n=8, INSL5<100 pg/mL n=14). (**C**) Interaction plot showing mean and 95% CI for change in stool consistency in response to placebo and ondansetron per INSL5 level for n=50 participants with bowel habit data available at baseline and week 12. BSFS, Bristol Stool Form Scale; IBS-D, irritable bowel syndrome with diarrhoea; INSL5, insulin-like peptide 5; TRITON study, abbreviation for previously performed clinical trial.

We assessed whether INSL5 levels influenced responsiveness to ondansetron treatment in the TRITON study, using an ANCOVA model examining effects of treatment (placebo or ondansetron) INSL5 group (above or below 100 pg/mL), and baseline stool consistency on 12-week BSFS. The overall model was significant (*F*(4,45)=4.36, p=0.005) and showed a statistically significant interaction term between treatment allocation and INSL5 grouping (p=0.025). A sensitivity analysis on the p value of this interaction term supported the use of a cut-off of 100 pg/mL to separate the high and low INSL5 groups (online supplemental figure 6A). These results suggest that patients with an INSL5>100 pg/mL had a more significant improvement in stool consistency when treated with ondansetron than patients with low INSL5 (figure 7A–C). A similar analysis was performed for PYY, but no threshold on sensitivity analysis produced a statistically significant interaction term between PYY group and treatment allocation (online supplemental figure 6B).

### INSL5 and PYY levels across all participant groups

Across all participants (n=101), there was a significant association between INSL5 and patient group (p=0.001), with increased INSL5 in patients with BAD versus patients with IBS-D (p_adj_=0.01) and healthy volunteers (p_adj_<0.001) (online supplemental figure 7A). There was also a small but significant relationship between BSFS and INSL5 (online supplemental figure 7B), p=0.007, r^2^=0.08, n=101).

Similarly, there was a significant association between PYY and patient group (n=88, p=0.001), with higher PYY in patients with BAD versus IBS-D (p_adj_<0.001) or healthy volunteers (p_adj_<0.001) (online supplemental figure 7C). Across all participants, BSFS did not correlate with PYY (online supplemental figure 7D, p=0.121, n=88).

## Discussion

This is the first study to assess INSL5 concentrations in patients with chronic diarrhoea, using a new immunoassay validated by mass spectrometry. Healthy volunteers with normal bowel habit had low INSL5, whereas patients with primary or secondary BAD and a subset of patients with IBS-D had elevated INSL5, with some evidence that INSL5 levels correlated with stool consistency on the BSFS, particularly in patients with BAD.

In a blinded study on seven healthy volunteers, rectal delivery of TCA evoked a prompt elevation of INSL5, building on previous in vitro studies which showed that GPBAR1 agonists including TCA can stimulate secretion of INSL5, PYY and GLP-1.^6 30^ Together with the finding of a correlation between fasting serum C4 and INSL5 levels, these results support the idea that elevated INSL5 levels seen in BAD arise directly from high luminal concentrations of bile acids stimulating L-cells in the distal colon and rectum. Although the finding of raised INSL5 in patients with BAD might suggest that elevated INSL5 levels could be a circulating biomarker for BAD, a subset of patients with IBS-D in whom a diagnosis of BAD had been excluded, also had elevated INSL5 concentrations.

The results highlight an association between INSL5 release and the motility response to rectal bile acid delivery, as INSL5 measurements were inversely correlated with the time interval before defecation post-enema and positively correlated with desire to defecate. Across all participants in the study, including healthy volunteers and patients with chronic diarrhoea, there was evidence of looser stools in patients with elevated INSL5 levels, although the r^2^ for the correlation between INSL5 levels and stool consistency was higher in patients with BAD (r^2^=0.35, p=0.001) than IBS-D (r^2^=0.06, p=0.033), with the latter of questionable significance given the small r^2^. The correlation observed in patients with BAD is consistent with a model in which elevated INSL5 arises from high levels of colonic bile acids, and diarrhoea results from either the high INSL5 (triggering increased motility), the bile acids themselves (eg, enhancing epithelial electrolyte secretion) or a combination of both. Further studies are needed to understand the stimuli responsible for raised INSL5 in some patients with IBS-D. Bile acids seem to explain only a small proportion of this variation, as there was only a weak relationship between faecal lithocholic acid and serum INSL5. Although secondary bile acids are strong GPBAR1-dependent stimuli of colonic L-cells,^30^ further work will be required to explore the contribution of other colonic metabolites such as microbially-generated SCFA, which stimulate INSL5 secretion in vitro^6^ and are known to increase with accelerated gut transit.^31^

The mechanism underlying the promotility effect of INSL5 remains incompletely understood. The INSL5 receptor RXFP4 is expressed by 5-HT producing enterochromaffin cells in the large intestine, which is interesting as antagonism of 5-HT3 receptors prevented the ability of an INSL5 analogue to accelerate rectal bead expulsion in mice.^7^ In this context, it is also noteworthy that the current study found that patients with IBS-D with raised INSL5 had additional clinical benefit in response to ondansetron, as shown by an improvement in average stool BSFS, although limited by a small number of participants. Although it is tempting to speculate that INSL5 might stimulate enterochromaffin cell 5-HT release which in turn increases intestinal motility, it is unlikely to be this simple as INSL5 has been linked to decreased, rather than increased, 5-HT release in vitro.^32^

In healthy volunteers receiving a bile acid enema and patients with confirmed BAD, a much stronger correlation was observed between INSL5 and PYY than between INSL5 and total GLP-1. However, fasting total GLP-1 levels should be interpreted with caution as the assay is known to cross-react with other proglucagon-derived peptides from pancreatic alpha-cells, which are particularly high in the fasting state.^33^ The relatively strong correlation between PYY and INSL5 in BAD is not unexpected, as ‘escape’ of bile acids from ileal uptake in BAD simultaneously impairs ileal FGF19 release, (reducing feedback inhibition on hepatic bile acid production measured by C4) and delivers more bile acids to PYY-producing L-cells in the colon, and INSL5-producing cells in the distal colon and rectum. Elevated PYY is unlikely itself to be causative of the diarrhoea in BAD and IBS-D, as PYY is known to have antisecretory and antipropulsory activity in the colon,^34^ contrasting with propopulsory activity of INSL5 in mice.^7^ Elevated PYY might, however, be a potential biomarker for BAD, especially as it was less elevated in IBS-D. The (admittedly weak) association of INSL5 but not PYY with BSFS in the TRITON IBS-D participants further supports a role of INSL5 in causing loose stools, although elevated INSL5 levels might simply reflect increased rectal epithelial exposure to luminal stimulants.

The absence of detectable INSL5 following food ingestion in healthy participants, or of a postprandial INSL5 response in participants with detectable fasting INSL5, likely reflects that *INSL5* is expressed only in the distal colon which does not normally receive ingested nutrients in the 3 hours following a meal.^3^,^5^ This supports the idea that L-cells are predominantly activated by the arrival of stimuli via the intestinal lumen, rather than by a proximal to distal neuronal circuit.^1 35^ It will be interesting in future studies to assess if and how INSL5 levels change around the time of defaecation when the rectum experiences rapidly changing exposure to luminal stimuli.

INSL5 levels measured using this immunoassay were considerably lower than those reported in previous studies^23 24^ which were typically>1000 pg/mL in all participants, while in the current study healthy volunteers had values<100 pg/mL and only patients with chronic diarrhoea had concentrations above this. The finding that INSL5 concentrations were similar when measured using the ELISA and LC-MS/MS argues against common assay problems, such as cross-reacting agents contributing substantially to the ELISA signal. INSL5 concentrations measured in this study are more in line with concentrations of other gut hormones, raising the possibility that previously reported INSL5 levels in humans may be complicated by immunoassay cross-reactivity.

In conclusion, this study is the first to explore circulating INSL5 levels in patients with chronic diarrhoea, highlighting a link between INSL5 and rectal bile acids, and revealing that INSL5 concentrations are elevated and associated with diarrhoea severity in BAD. Additionally, INSL5 was raised in some patients with IBS-D, with the observation that those patients had a better clinical response to ondansetron. Further exploration is required to determine whether increased INSL5 secretion directly mediates increased intestinal motility in chronic diarrhoea; whether raised INSL5 concentrations could be used as a biomarker for the diagnosis of colonic and rectal irritation or inflammation; and the potential utility of ondansetron for symptom relief in patients with chronic diarrhoea, particularly in people with elevated INSL5. It will also be interesting to explore whether RXFP4 could be developed as a target for the treatment of conditions with altered colonic motility such as BAD.

## Supplementary material

10.1136/gutjnl-2025-335393 online supplemental file 1

10.1136/gutjnl-2025-335393 online supplemental file 2

## Data Availability

Data are available upon reasonable request.
