# Supplementary material for: Insulin-like peptide 5 is released in response to bile acid in the rectum and is associated with diarrhoea severity in patients with bile acid diarrhoea
Source: Gut. 2025 Jul 23;75(2):e335393. doi: 10.1136/gutjnl-2025-335393 (PMC7618615; doi:10.1136/gutjnl-2025-335393)
Supplement: online supplemental file 2 [file gutjnl-75-2-s002.pptx]

## Slide 1
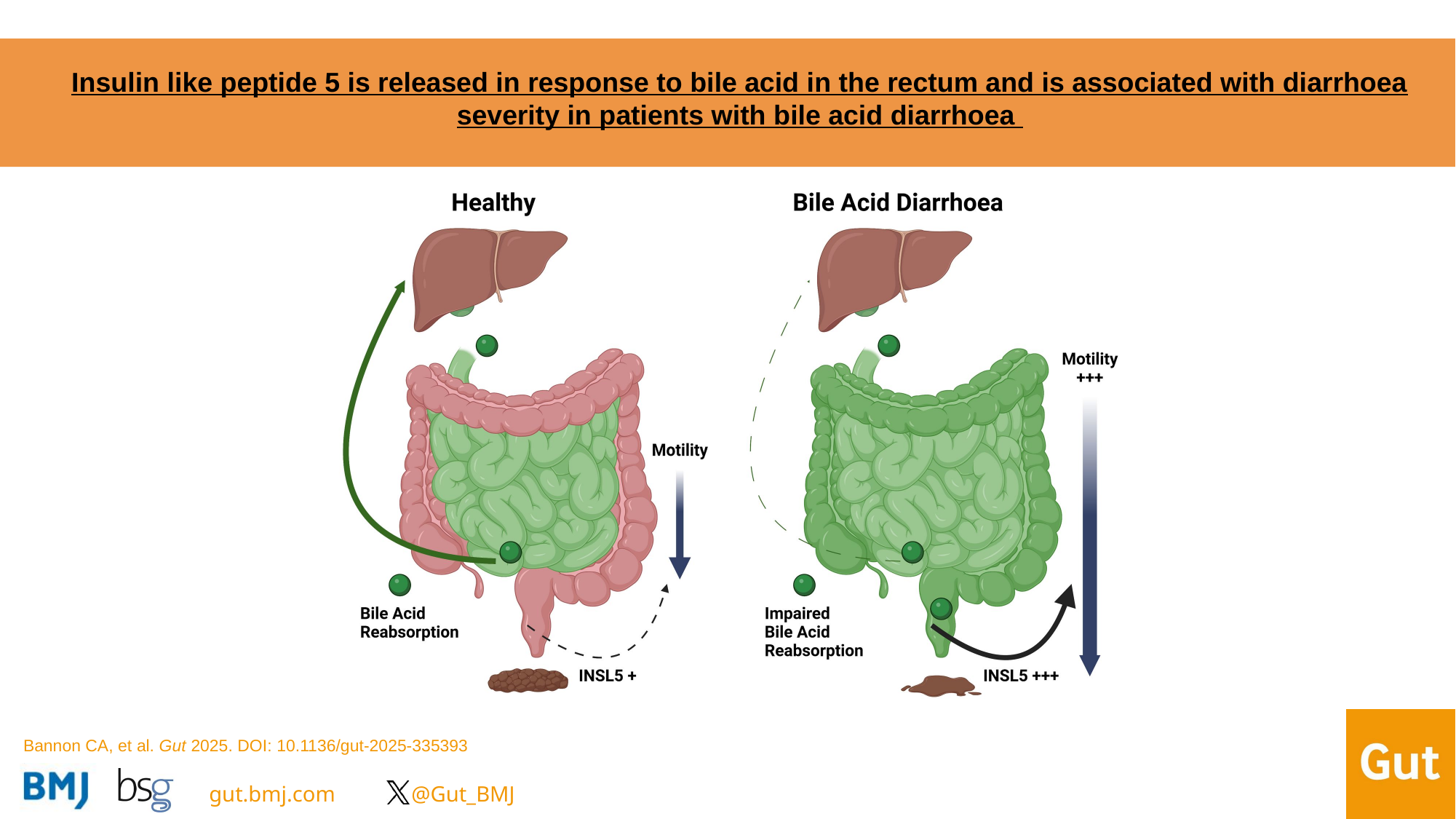

Insulin like peptide 5 is released in response to bile acid in the rectum and is associated with diarrhoea severity in patients with bile acid diarrhoea
Bannon CA, et al. Gut 2025. DOI: 10.1136/gut-2025-335393
gut.bmj.com
@Gut_BMJ
