# Supplementary material for: Insulin like peptide 5 is released in response to bile acid in the rectum and is associated with diarrhoea severity in patients with bile acid diarrhoea
Source: Gut. Author manuscript; Available in PMC 2026 Jan 17. (PMC7618615; doi:10.1136/gutjnl-2025-335393)
Supplement: Supplementary file 1 [file EMS211982-supplement-Supplementary_Materials.pdf]

Insulin like peptide 5 is released in response to bile acid in the rectum and is associated with diarrhoea severity in patients with bile acid diarrhoea

Supplementary Figures:

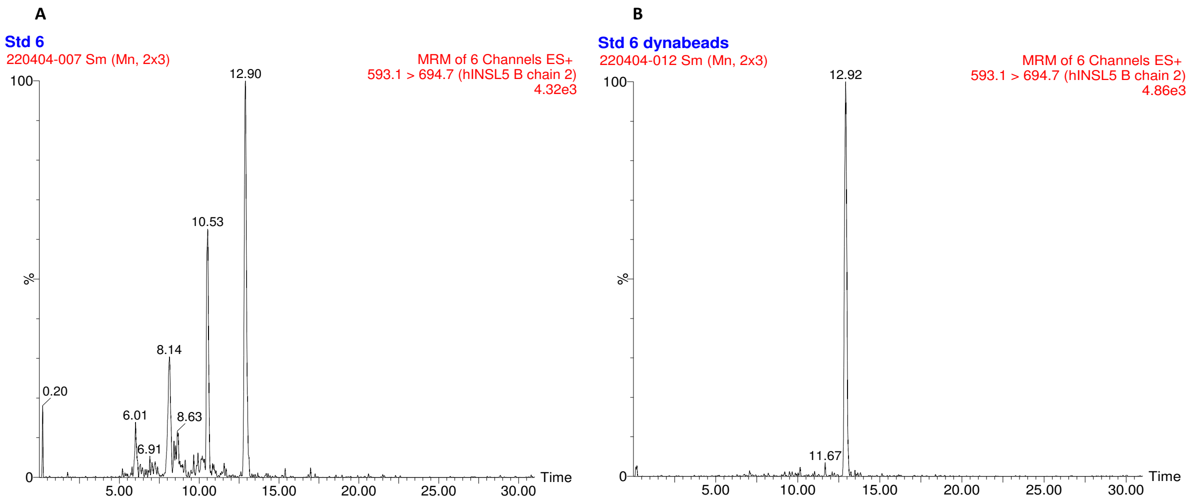

Supplementary figure 1:

A: Synthetic INSL5 was spiked into pooled human plasma, and then run on LC-MS/MS after acetonitrile precipitation

B: The same sample was analysed after immunoprecipitation extraction using the antibody in the immunoassay.

Acetonitrile extraction of spiked INSL5 had same recovery when extracted using immunoprecipitation with the INSL5 antibody in the immunoassay, showing antibody is specific and binds to INSL5.

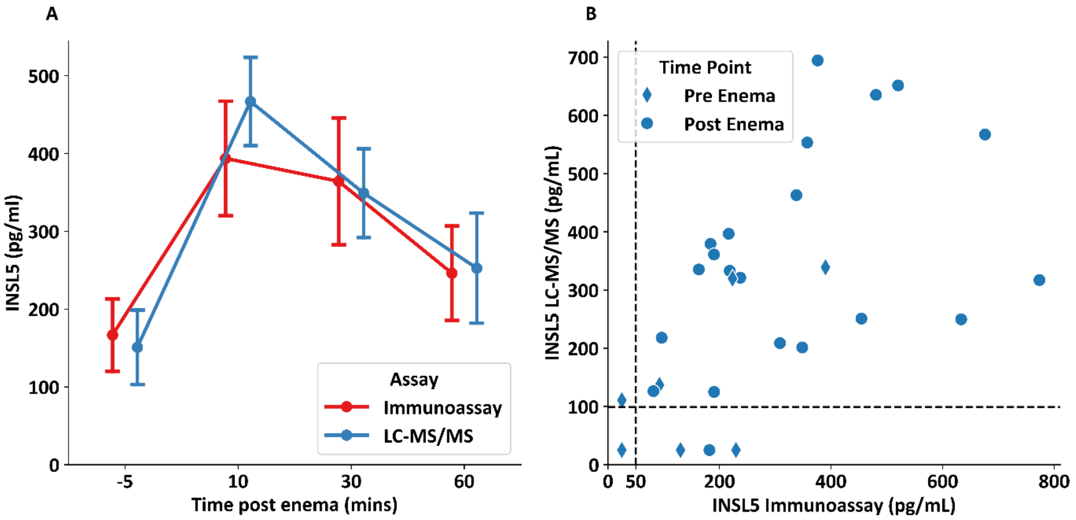

Supplementary figure 2: INSL5 measured via LC-MS/MS in participants receiving a 3500mg TCA enema, compared with INSL5 measurements from immunoassay

A – Point plot of INSL5 measurements from plasma samples from visit where each participant received 3500mg rectal TCA enema

B – Scatterplot of INSl5 quantification via immunoassay and LC-MS/MS for samples from 3500mg TCA enema visit indicating similar concentrations measured with each technique.

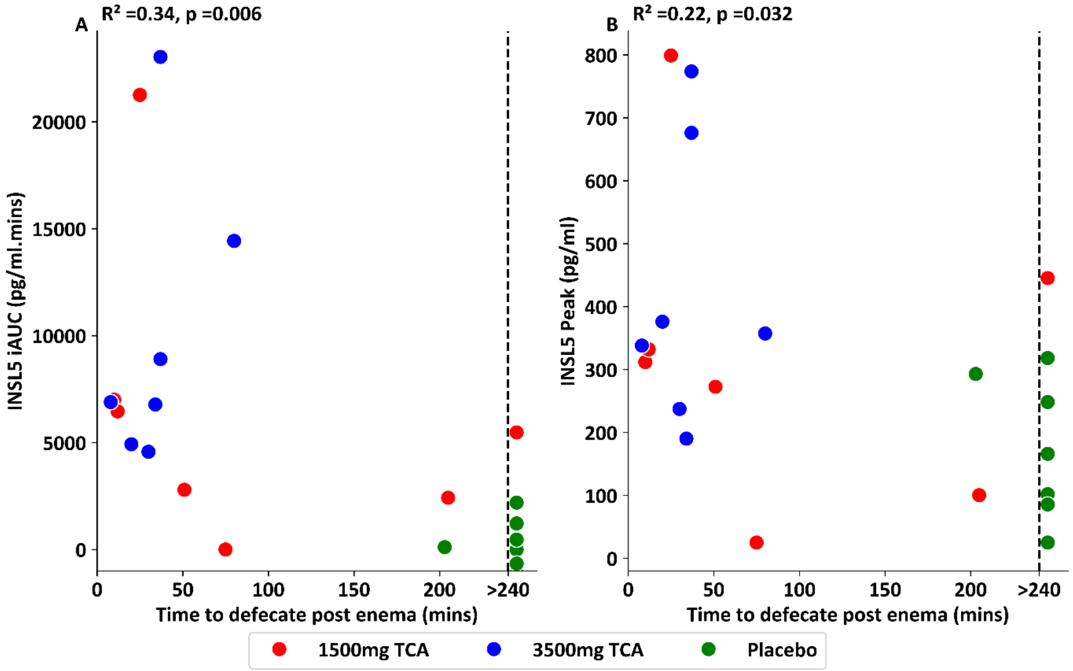

Supplementary figure 3: Comparison of INSL5 levels to time to defecate

A: Scatterplot showing the iAUC of INSL5 level post enema vs the time to defecate post enema. Linear regression performed to compare INSL5 delta vs time to defecate, showing evidence of a negative correlation (p=0.006, r^2 = 0.34).

B: Scatterplot showing the peak INSL5 level post enema vs the time to defecate post enema. Linear regression performed to compare INSL5 delta vs time to defecate, showing evidence of a negative correlation (p=0.032, r^2 = 0.22).

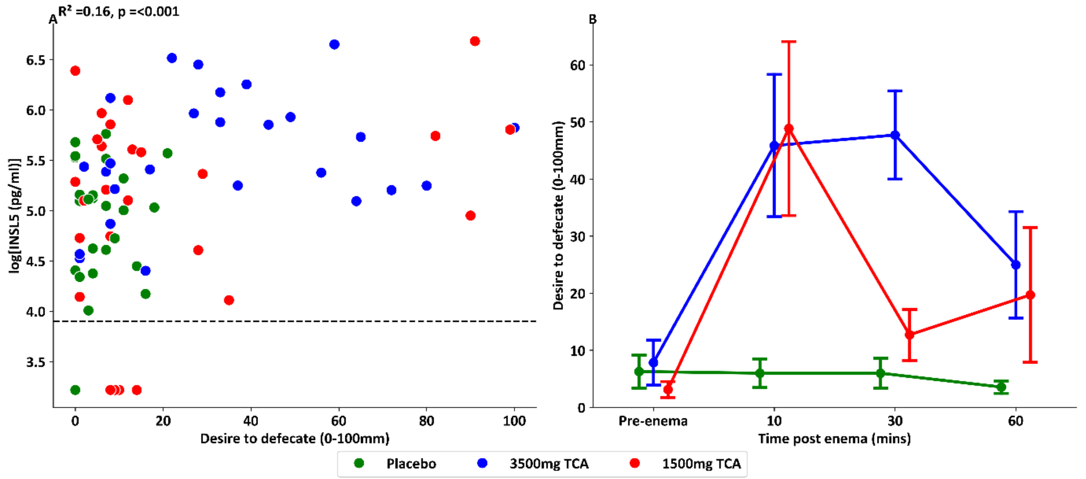

Supplementary figure 4: Comparison of INSL5 to desire to defecate post enema

A: Scatterplot of log transformed INSL5 at each time point to desire to defecate score (measurement to number of mm marked on a 0 – 100mm visual analogue scale), (significant evidence of correlation: $p<0.001$ $r2 = 0.16$).

B: Point plot of visual analogue score of desire to defecate pre and post enema.

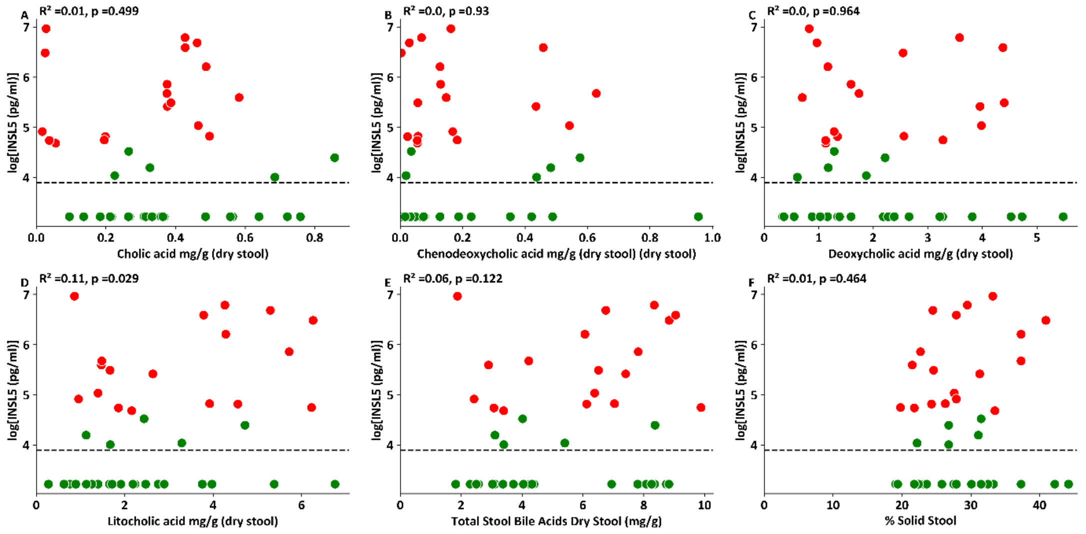

Supplementary Figure 5: Comparison of natural logarithm transformed baseline serum INSL5 level in IBS-D samples from the TRITON study and paired collected faecal bile acid levels in n=44 participants.

Note – dotted line denotes limit of detection of INSL5 of 50 pg/ml.

A: Comparison of INSL5 level to cholic acid (primary bile acid) (no significant evidence of correlation).

B: Comparison of INSL5 level to chenodeoxycholic cholic acid (primary bile acid) (no significant evidence of correlation).

C: Comparison of INSL5 level to deoxycholic acid (secondary bile acid) (no significant evidence of correlation).

D: Comparison of INSL5 level to lithocholic acid (secondary bile acid) (significant evidence of correlation: p=0.029 r2 = 0.11).

E: Comparison of INSL5 level to total stool bile acids (as measured as 4 stool bile acid metabolites added together) (no significant evidence of correlation).

F: Comparison of INSL5 level to percentage that stool sample was solid (no significant evidence of correlation).

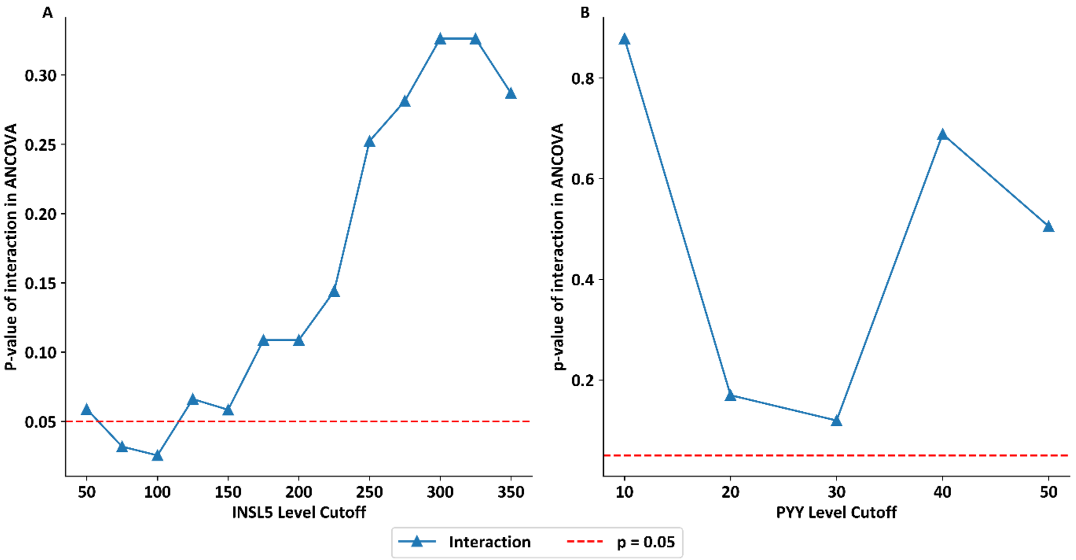

Supplementary figure 6: ANCOVA sensitivity analysis for INSL5 group cut off for Triton IBS-D Samples

ANCOVA model was used with BSFS at week 12 as the dependent variable, treatment allocation (Active vs Placebo) and hormone group (above or below threshold) as categorical variables and baseline BSFS at enrolment and interaction between treatment allocation and INSL5 group as covariates

A: Sensitivity analysis to assess p-value in ANCOVA interaction term between treatment allocation (placebo or ondansetron) and INSL5 group, using different cut offs for INSL5 group – analysis supports a cut off of 100 pg/ml.

B: Sensitivity analysis to assess p-value in ANCOVA interaction term between treatment allocation (placebo or ondansetron) and PYY group, using different cut offs for PYY group – no cut off to make a high and low level PYY group observed.

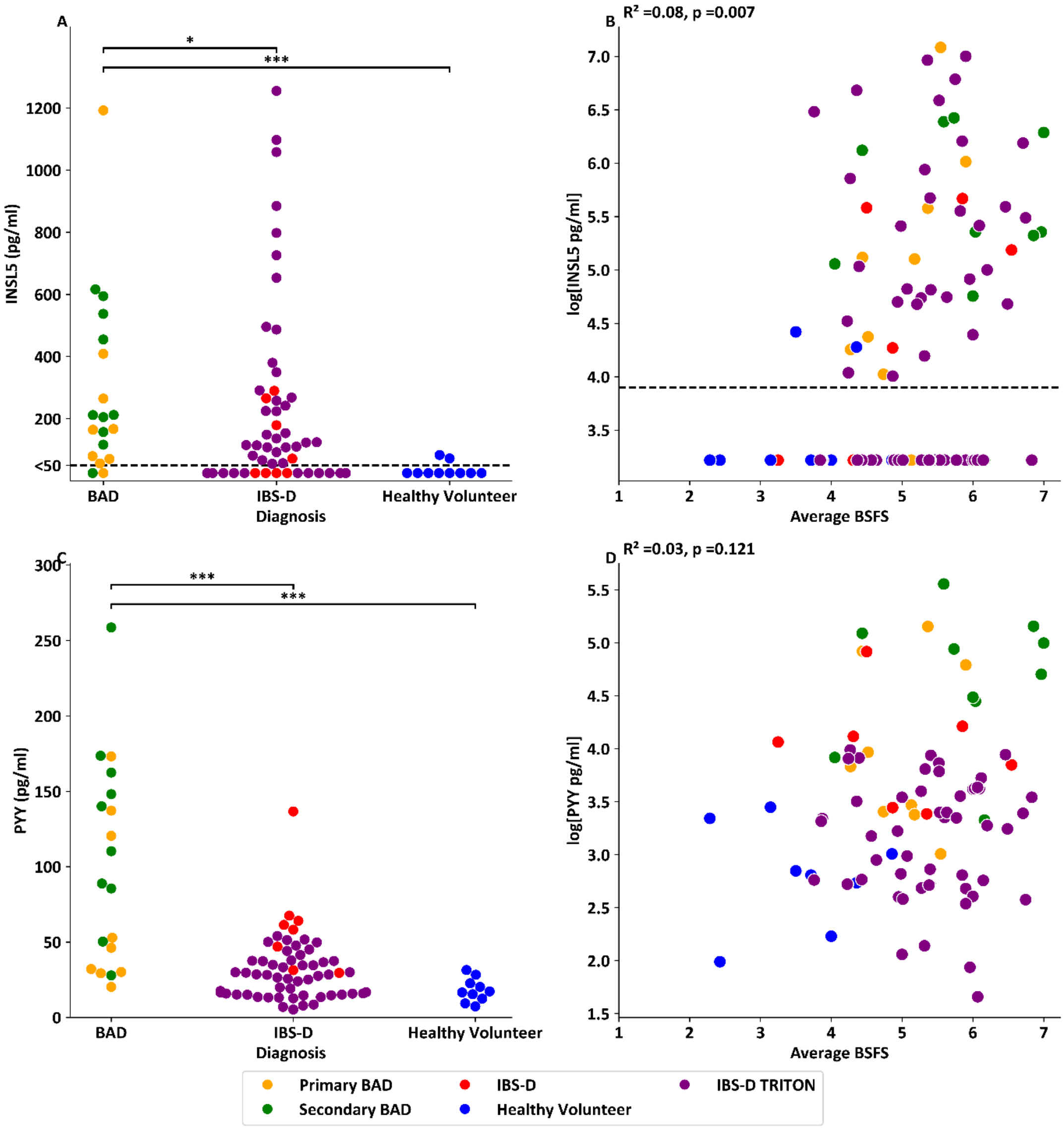

Supplementary Figure 7: INSL5 and PYY levels from all samples and compared to BSFS

A: Swarm plot showing fasting INSL5 levels in healthy volunteers (n=10), patients with BAD (n=19), IBS-D (n=72). Dotted line denotes immunoassay sensitivity limit of 50 pg/ml.

An ANCOVA model performed on natural logarithm transformed data with age and gender as co-variates, showed a significant main effect of diagnosis (F(2, 96) =6.84, p = 0.002).

Tukey HSD test for multiple comparisons on natural logarithm transformed data, showed statistically significant differences between patients with BAD and healthy volunteers (p<0.001) and patients with BAD and patients with IBS-D (p=0.012).

B: Scatterplot showing natural logarithm transformed INSL5 levels vs average BSFS from bowel habit chart (p=0.007, r^2 = 0.08) (IBS-D n=69; BAD n=19, healthy volunteer n=8).

C: Swarm plot showing fasting PYY levels in healthy volunteers (n=10), patients with BAD (n=19), and IBS-D (n=59).

An ANCOVA model performed on natural logarithm transformed data with age and gender as co-variates, showed a significant main effect of diagnosis ($F(2,83) = 25.2$, $p < 0.001$).

Tukey HSD test for multiple comparisons on natural logarithm transformed data, showed statistically significant differences between patients with BAD and healthy volunteers ($p<0.001$) and patients with BAD and patients with IBS-D ($p<0.001$).

D: Scatterplot showing natural logarithm transformed PYY levels vs average BSFS from bowel habit chart ($p=0.121$, $r^2 = 0.03$) (IBS-D n=58; BAD n=19, healthy volunteer n=8).

Supplementary Data

Table 1. ANCOVA results for INSL5 levels, comparing patient groups from samples from Walters et al and Foreman et al.

Predictor	Sum of Squares	df	F	p-value
Diagnosis	19.31	3	7.01	0.0010
Gender	0.07	1	0.08	0.7848
Age	0.02	1	0.02	0.8882
BMI	2.41	1	2.63	0.1155
Residual	27.56	30	–	–

Table 2. ANCOVA results for PYY levels, comparing patient groups from samples from Walters et al and Foreman et al.

Predictor	Sum of Squares	df	F	p-value
Diagnosis	16.93	3	18.66	<0.0001
Gender	1.38	1	4.56	0.0411
Age	0.92	1	3.04	0.0912
BMI	1.05	1	3.46	0.0726
Residual	9.08	30	–	–

Table 3. Pairwise group comparisons on log-transformed data for fasting INSL5 levels comparing patient groups from samples from Walters et al and Foreman et al.

Group 1	Group 2	Mean Difference	Adjusted p-value	95% CI (Lower – Upper)	Significant
Healthy Volunteer	IBS-D	0.75	0.3632	–0.48 to 1.98	No
Healthy Volunteer	Primary BAD	1.53	0.0077	0.34 to 2.72	Yes
Healthy Volunteer	Secondary BAD	1.98	0.0003	0.82 to 3.14	Yes
IBS-D	Primary BAD	0.78	0.3577	–0.48 to 2.04	No

IBS-D	Secondary BAD	1.23	0.0501	–0.00 to 2.46	No
Primary BAD	Secondary BAD	0.45	0.7329	–0.74 to 1.65	No

Table 4. Pairwise group comparisons on log-transformed data for fasting PYY levels comparing patient groups from samples from Walters et al and Foreman et al.

Group 1	Group 2	Mean Difference	Adjusted p-value	95% CI (Lower – Upper)	Significant
Healthy Volunteer	IBS-D	1.21	0.0010	0.43 to 1.99	Yes
Healthy Volunteer	Primary BAD	1.19	0.0009	0.43 to 1.94	Yes
Healthy Volunteer	Secondary BAD	1.86	<0.0001	1.12 to 2.59	Yes
IBS-D	Primary BAD	–0.03	0.9997	–0.83 to 0.77	No
IBS-D	Secondary BAD	0.64	0.1363	–0.14 to 1.43	No
Primary BAD	Secondary BAD	0.67	0.0975	–0.09 to 1.43	No

Table 6. ANCOVA Results for Consistency at Week 12 in samples from the TRITON study

Predictor	Coefficient (β)	Std. Error	t-value	p-value	95% CI (Lower – Upper)
Intercept	0.663	1.194	0.56	0.581	–1.74 to 3.07
Allocation (Ondansetron vs Placebo)	0.017	0.432	0.04	0.969	–0.85 to 0.89
INSL5 Group (>100 pg/ml vs ≤100 pg/ml)	0.273	0.430	0.64	0.528	–0.59 to 1.14
Interaction: Allocation × INSL5 Group	–1.517	0.657	–2.31	0.025	–2.84 to –0.20
Baseline consistency	0.700	0.227	3.08	0.003	0.24 to 1.16

Model Summary

- **R^2:** 0.279
- **Adjusted R^2:** 0.215
- **F-statistic:** 4.36 (p = 0.0046)
- **Number of observations:** 50
- **AIC:** 158.5
- **BIC:** 168.1
- **Durbin-Watson:** 1.89

Table 7. ANCOVA Results for INSL5 levels across all samples (Adjusted for Gender and Age)

Source	Sum of Squares	df	F	p-value
Diagnosis	19.51	2	6.84	0.0017
Gender	0.50	1	0.35	0.5562
Age	0.05	1	0.03	0.8553
Residual	136.85	96	—	—

Table 8. ANCOVA Results for PYY (Adjusted for Gender and Age)

Source	Sum of Squares	df	F	p-value
Diagnosis	21.09	2	25.18	2.83×10^{-9}
Gender	0.96	1	2.30	0.1335
Age	0.11	1	0.26	0.6098
Residual	34.76	83	—	—

Table 9. Pairwise Comparisons on Log-Transformed INSL5 per participant group across all samples

Group 1	Group 2	Mean Difference	95% CI (Lower – Upper)	Adjusted p-value	Significant
BAD	Healthy Volunteer	–1.77	–2.87 to –0.67	0.0007	Yes
BAD	IBS-D	–0.90	–1.62 to –0.17	0.0115	Yes
Healthy Volunteer	IBS-D	0.87	–0.08 to 1.82	0.0793	No

Table 10. Pairwise Comparisons on Log-Transformed INSL5 per participant group across all samples

Group 1	Group 2	Mean Difference	95% CI (Lower – Upper)	Adjusted p-value	Significant
BAD	Healthy Volunteer	–1.54	–2.14 to –0.93	<0.0001	Yes
BAD	IBS-D	–1.08	–1.49 to –0.67	<0.0001	Yes
Healthy Volunteer	IBS-D	0.46	–0.07 to 0.99	0.1014	No
